# WHAT FACTORS ARE ASSOCIATED WITH SELF-PERCEPTION OF INTENSE FAMILY CAREGIVING?

**DOI:** 10.1093/geroni/igae098.2148

**Published:** 2024-12-31

**Authors:** Susan Hassell, Joanne Campione

**Affiliations:** Westat, Rockville, Maryland, United States; Westat, Rockville, Maryland, United States

## Abstract

The stress and anxiety associated with family caregiving can contribute to the caregiver’s own health and well-being and can also be linked to less effective caregiving. As part of the 2023 National Survey of Older Americans Act (OAA) Participants, the following item was asked among 1,187 clients of the National Family Caregiver Support Program (NFCSP): “On a scale of 1 to 5, where 5 is very intense, how intense is the care you provide?” Fifty-one percent responded that the care is intense or very intense. Of the remaining caregivers, 15% responded that the care was not intense and 34% felt neutral. We first tested 62 survey variables for bivariate significant differences between “intense care” vs. “not intense care” and then used stepwise multivariable logistic regression. Variables included caregiver demographics, caregiving situational factors, caregiver nutrition, caregiver well-being, care receiver (CR) demographics and health conditions, and the number of activities of daily living (ADL) difficulties experienced by the CR. From the final logistic regression model (c-statistic = 0.74), the factors associated with a significant increase (p < 0.01) in the likelihood of self-reported intense care were: 1) CR’s number of ADL difficulties, 2) caregiver food insecurity score, 3) CR has Alzheimer’s or other memory-related disease, 4) caregiver provides all or almost all the care, and 5) the caregiver has no time for self. These findings provide a deeper understanding of the type of caregivers that may need more support from the NFCSP and from the OAA’s case management and home-delivered meals programs.

